# SARS-CoV-2 Variant Delta Potently Suppresses Innate Immune Response and Evades Interferon-Activated Antiviral Responses in Human Colon Epithelial Cells

**DOI:** 10.1128/spectrum.01604-22

**Published:** 2022-09-08

**Authors:** Dixit Tandel, Vishal Sah, Nitesh Kumar Singh, Poojitha Sai Potharaju, Divya Gupta, Sauhard Shrivastava, Divya Tej Sowpati, Krishnan H. Harshan

**Affiliations:** a CSIR-Centre for Cellular and Molecular Biology, Hyderabad, Telangana, India; b Academy for Scientific and Innovative Research (AcSIR), Ghaziabad, Uttar Pradesh, India; UC Davis

**Keywords:** SARS-CoV-2, COVID-19, variants, Delta, RLR pathway, innate antiviral

## Abstract

The Delta variant of SARS-CoV-2 has caused more severe infections than its previous variants. We studied the host innate immune response to Delta, Alpha, and two earlier variants to map the evolution of the recent ones. Our biochemical and transcriptomic studies in human colon epithelial cell line Caco2 reveal that Alpha and Delta have progressively evolved over the ancestral variants by silencing the innate immune response, thereby limiting cytokine and chemokine production. Though Alpha silenced the retinoic acid-inducible gene (RIG)-I-like receptor (RLR) pathway just like Delta did, it failed to persistently silence the innate immune response, unlike Delta. Both Alpha and Delta have evolved to resist interferon (IFN) treatment, while they are still susceptible to RLR activation, further highlighting the importance of RLR-mediated, IFN-independent mechanisms in restricting SARS-CoV-2. Our studies reveal that SARS-CoV-2 Delta has integrated multiple mechanisms to silence the host innate immune response and evade the IFN response. We speculate that Delta’s silent replication and sustained suppression of the host innate immune response, thereby resulting in delayed or reduced intervention by the adaptive immune response, could have potentially contributed to the severe symptoms and poor recovery index associated with it. It is likely that this altered association with the host would play an important role in the coevolution of SARS-CoV-2 with humans.

**IMPORTANCE** Viruses generally learn to coexist with the host during the process of evolution. It is expected that SARS-CoV-2 would also evolve to coexist in humans by trading off its virulence for longer persistence, causing milder disease. Clinically, the fatality associated with COVID-19 has been declining due to vaccination and preinfections, but the Delta variant caused the most severe disease and fatality across several parts of the world. Our study identified an evolving trend of SARS-CoV-2 variants where the variants that emerged during early parts of the pandemic caused a more robust innate immune response, while the later emerging variant Delta showed features of suppression of the response. The features that Delta has acquired could have strongly influenced the distinct pathophysiology associated with its infection. How these changed associations with the host influence the long-term evolution of the virus and the disease outcome should be closely studied to understand the process of viral evolution.

## INTRODUCTION

SARS-CoV-2, the causative virus behind the current COVID-19 pandemic, has been evolving since its first detection in humans in 2019 (1), generating newer variants with higher infectivity (2). Delta (B.1.617.2), a dominant variant of concern (VOC) with higher severity (3, 4), had successfully outgrown the other variants (5) and caused several breakthrough infections (6). The newest VOC, Omicron, has caused major waves of infection across the world, but with significantly lower severity than Delta (7, 8). Before Delta, Alpha (B.1.1.7), another VOC, had higher transmissibility than its contemporary variants (3). Though variants such as Beta (B.1.351) and Gamma (P.1) were considered potential VOCs at one time, they failed to dominate across the world. Immune evasion by the new variants against the antibodies generated against the previous variants or vaccines is natural during viral evolution and has been the case for Delta (9, 10). Though a trade-off between the virulence and transmissibility has been evident in several viral infections, there are exceptions as well (11). It is unclear if the subsequent SARS-CoV-2 variants have been adapting in humans and causing more benign infections.

It is now fairly well understood that humoral immune escape coupled with increased transmissibility are important factors for a particular variant to gain dominance in the pandemic (12). Increased transmissibility is rendered by several factors, including enhanced entry and better survival. Epithelial cells in the respiratory and intestinal systems are permissive to SARS-CoV-2 both *in vitro* and *in vivo* (13). The innate immune response instructs the adaptive response through cytokines, chemokines, and antigen presentation (14). There is no conclusive evidence of a productive infection of immune cells by SARS-CoV-2 (15, 16). The cytokine storm that has been implicated in the severe COVID-19 symptoms (17, 18) is an outcome of excessive secretion of proinflammatory cytokines first secreted by the epithelial cells and in response by dendritic cells (DC) and other immune cells.

The retinoic acid-inducible gene (RIG)-I-like receptor (RLR) pathway constitutes an important network recognizing double-stranded RNA (dsRNA) intermediates of RNA viruses (19). Both RLR and Toll-like receptor (TLR) pathways are significantly impaired or delayed in COVID-19 patients (20–22) and validated in epithelial culture models (23, 24), contributing to COVID pathogenicity (18). Production of type-I interferons (IFNs) and subsequent activation of the JAK-STAT pathway are targeted by viral proteins, and the Alpha variant has evolved better mechanisms to evade innate response (24, 25). With a hypothesis that the newer and more successful variants are better at suppressing the innate responses, we investigated the details of RLR pathway activation in response to five variants of SARS-CoV-2, including Delta. Our results demonstrate a steady progression in the capabilities of the subsequent variants over the previous ones in either delaying or efficiently suppressing the innate immune response. Delta suppressed the host response pathways RLR-IFN and JAK-STAT most successfully and also resisted IFN treatment. Gene expression analysis uncovered that Delta suppressed the host response in general, including all major innate immune response pathways, much more profoundly than Alpha, which itself was evidently more advanced than the previous variants. This suggested that Delta has been able to replicate in the host without alerting the innate signal pathways, and this could possibly have resulted in delayed activation of the adaptive response. Our findings could be important in the ever-changing contexts of COVID-19 symptoms and intervention strategies in addition to providing important clues to the evolutionary dynamics of SARS-CoV-2.

## RESULTS

### Delta genomic RNA has high replicative fitness in culture but generates low infectious viral titers.

**FIG 1:**
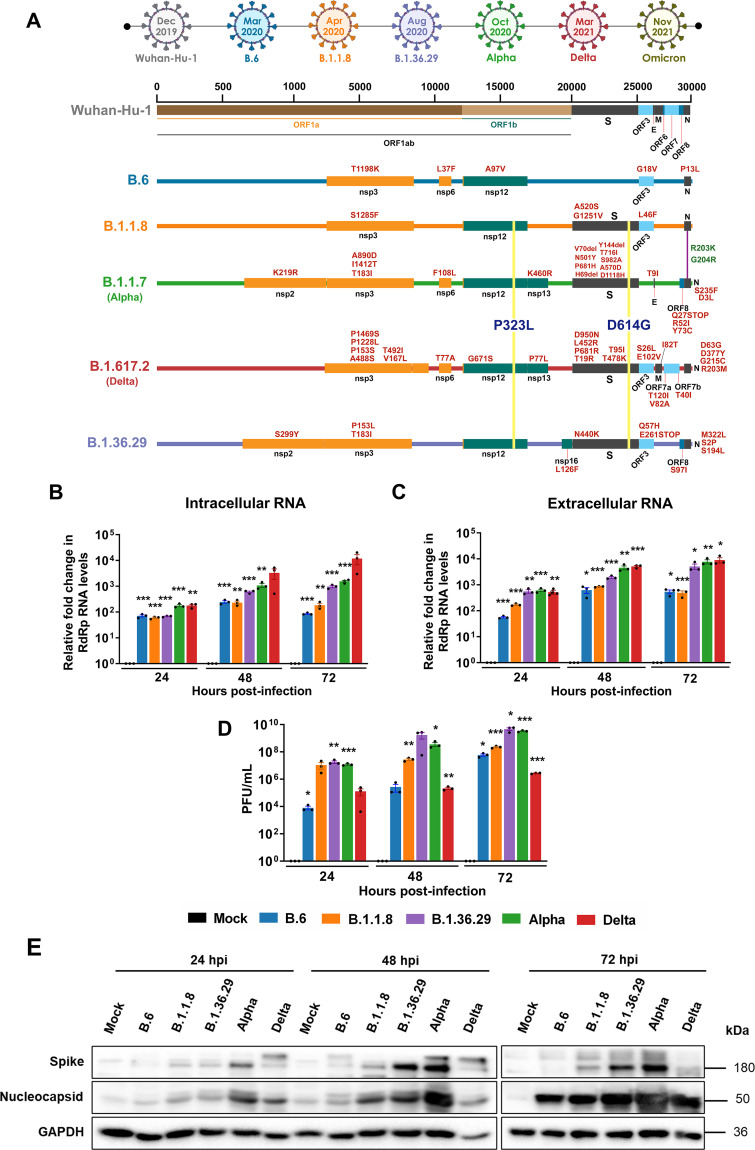
Delta has the highest RNA replication efficiency but also has low infectious titers. (A) Schematic representing the mutations found in the five distinct variant isolates compared with the ancestral Wuhan isolate. The timescale on the top represents the month of the first reporting of the variant in GISAID. (B) Intracellular SARS-CoV-2 RNA quantified by real-time qRT-PCR detection of the viral RdRp region. Caco2 cells were infected with 1 MOI of one of the five distinct variant isolates and incubated for the specific time intervals as shown in the graph. The cells were harvested, and RNA prepared, from which SARS-CoV-2 RNA was analyzed by real-time qRT-PCR. The fold changes against the mock-infected samples were generated through the ΔΔ*CT* method by normalizing against the internal control RNase P of the corresponding sample and have been plotted in the graph. Each color represents an individual variant as described in the common color legend provided in panel D. (C) SARS-CoV-2 RNA in the culture supernatants quantified by real-time qRT-PCR detection of the viral RdRp region. As in panel B, the fold changes in the levels were plotted against the mock-infected samples for individual time points and normalized against RNase *P* values. (D) Infectious titers of SARS-CoV-2 from culture supernatants infected with the distinct variant isolate, determined by PFA. The culture supernatants collected at specific time intervals postinfection were cleared of debris and were serially diluted and used as inoculum to infect fresh monolayers of Vero cells. The infected wells were layered with agarose, and the plaques formed were identified by staining with crystal violet. Raw values were plotted as PFU/mL for specific time intervals and individual variants. All the graphs contain results from biological triplicates. (E) Immunoblots detecting the levels of SARS-CoV-2 S and N proteins in the cells infected with the respective variant at the specific time interval. A common color legend describing the variant identity has been provided in panel D. All graphs were prepared using GraphPad Prism version 8.0.2. Statistical significance is represented as *, **, and *** for *P* < 0.05, *P* < 0.01, and *P* < 0.005, respectively.

Since Delta and Alpha variants had higher infectivity in populations, we decided to compare their replicative and infectious fitness with the earlier variant isolates in time course experiments in Caco2 cells. Previous studies have demonstrated that Caco2 cells are highly permissive to SARS-CoV-2 (13). In a comparative analysis, both lung epithelial cell line Calu3 and colon epithelial cell line Caco2 showed comparable permissivity to SARS-CoV-2 (see Fig. S1A and B in the supplemental material). SARS-CoV-2 induced interferon regulatory factor 3 (IRF3) phosphorylation in Caco2 (as shown in the upcoming section), but not in Calu3 (Fig. S1C), thus suggesting its suitability in our studies. Colon epithelium is a target of SARS-CoV-2, and intestinal distress being a major symptom in COVID-19, the choice of Caco2 is relevant to this study. Cells were infected with 1 multiplicity of infection (MOI) of five SARS-CoV-2 variant isolates (B.6, B.1.1.8, B.1.36.29, B.1.1.7 [Alpha], and B.1.617.2 [Delta]) for up to 72 h postinfection (hpi), and the cellular and supernatant viral RNA titers and infectious titers were measured. Genetic variation among these variants is depicted in Fig. 1A. B.6 is an isolate of the A3i, clade which was prominent during the early part of the pandemic, while B.1.1.8 belongs to the A2a clade, which diverged with a characteristic D614G conversion in the spike protein (S). B.1.36.29, another isolate of the A2a clade, several cases of which were reported in India, has an additional characteristic N440K mutation in the RBD of S. Intracellular RNA analysis revealed that Delta replicated most efficiently right from 24 to 72 hpi (Fig. 1B), followed by Alpha, B.1.36.29, B.1.1.8, and B.6 in that order. Viral RNA levels in the supernatant followed a similar trend (Fig. 1C). However, the infectious titer data differed from the replication data, where Delta displayed the fewest titers with B.1.36.29, and Alpha attained the highest titers, followed by B.1.1.8, and B.6 (Fig. 1D). Thus, the higher rate of RNA replication of Delta did not translate into high infectious fitness. It is likely that the lower titers could be a result of a lower plaquing efficiency of Delta in Vero cells, as SARS-CoV-2 variants display variable tropism in cultured cells (26). The relatively lower infectious titers of Delta also suggested that viral load may not be a major factor behind its higher transmissivity. Interestingly, spike (S) and nucleocapsid (N) immunoblots revealed that Alpha and B.1.36.29 follow a pattern of high levels of S and N (Fig. 1E) that correlated with their infectious titers, indicating that the higher availability of the structural proteins could be a determining factor in their higher infectious titers.

### RLR and JAK-STAT are activated by early variants, but not by Delta.

**FIG 2:**
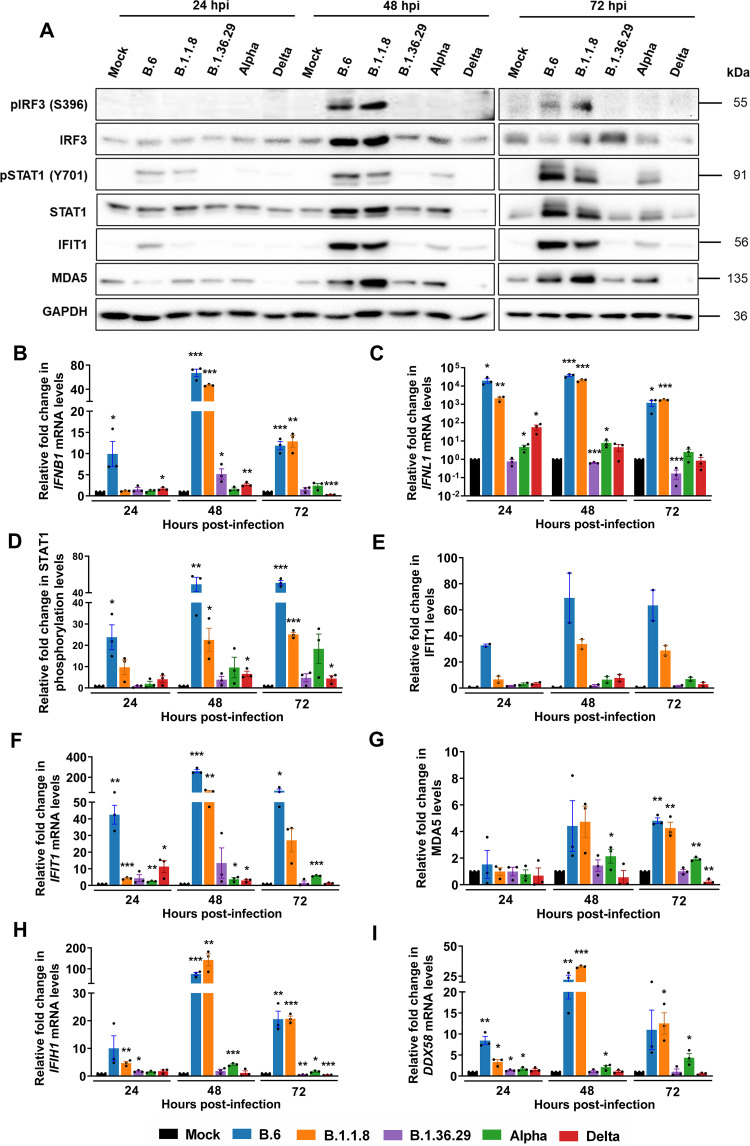
Delta infection causes long-term and complete suppression of the RLR and JAK-STAT pathways. (A) Immunoblot images demonstrating the phosphorylation of IRF3 and STAT1 along with the expression of ISGs IFIT1 and MDA5 in Caco2 cells infected separately with one of the five variant isolates. (B) qRT-PCR quantification of *IFNB1* transcripts in cells infected with the individual variants. (C) Similar quantification for *IFNL1* transcripts. (D) Densitometric quantification of the phosphorylation of STAT1 across the infected samples. (E and F) Densitometric quantification of IFIT1 (D) and qRT-PCR (E) analysis of its transcripts (*n* = 2). (G and H) Densitometric quantification of MDA5 expression (G) and qRT-PCR analysis (H) of its transcripts. (I) qRT-PCR quantification of *DDX58* transcripts in individual infections. All the graphs are representatives of biological triplicates. A common color legend describing the variant identity has been provided at the bottom of the figure. All graphs were prepared using GraphPad Prism version 8.0.2. GAPDH was used as the normalization control for qRT-PCR. Statistical significance is represented as *, **, and *** for *P* < 0.05, *P* < 0.01, and *P* < 0.005, respectively.

We next analyzed the RLR-mediated innate response to SARS-CoV-2 variants. IRF3 phosphorylation, a good measure of RLR activation, was activated by B.6 and B.1.1.8 variants by 48 hpi and continued until 72 hpi in Caco2 cells (Fig. 2A). However, Alpha, Delta, and B.1.36.29 failed to activate IRF3 phosphorylation throughout 72 hpi, indicating that they have employed additional mechanisms to completely silence RLR activation. *IFNB1* expression at 24 hpi was limited to B.6 infection (Fig. 2B), whereas by 48 hpi, strong induction was also found in B.1.1.8. Phenomenal induction of *IFNL1* by B.6 and B.1.1.8 from 24 hpi and at moderate levels by Alpha indicated that it is regulated distinctly from *IFNB1* (Fig. 2C). Intriguingly, Delta caused considerable induction of *IFNL1* at 24 hpi, which faded progressively with time. The induction of *IFNB1* and *IFNL1* in the absence of IRF3 phosphorylation suggested that they are activated by IRF3-independent mechanisms during SARS-CoV-2 infection. B.1.36.29 most successfully suppressed both *IFNB1* and *IFNL1* activation. STAT1 phosphorylation in B.6 and B.1.1.8 infections by 24 hpi confirmed IFN-mediated activation of the JAK-STAT pathway (Fig. 2A and D). Delta infection delayed STAT1 phosphorylation until 72 hpi, while Alpha induced a modest and steady phosphorylation after 24 hpi. Interferon-induced protein with tetratricopeptide repeats 1 (IFIT1) and its transcript levels closely mirrored STAT1 phosphorylation (Fig. 2A, E, and F). Similar inductions of melanoma differentiation associated protein 5 (MDA5) and its transcript *IFIH1* (Fig. 2G and H, respectively) and *DDX58* transcripts (Fig. 2I) further confirmed a strong activation of interferon-stimulated genes (ISGs) in B.6 and B.1.1.8 infections and modest induction in Alpha, but insignificant induction in Delta and B.1.36.29 infections. These results indicated that the earlier variants indeed caused delayed RLR activation, but the later variants Alpha and Delta, failed to activate the RLR pathway. It is intriguing, though, that B.1.36.29, which suppressed the RLR response more efficiently than Alpha, had emerged well before it but could not become a dominant variant.

### Delta and Alpha evade the IFN response but are partially susceptible to RLR activation by poly(I·C).

**FIG 3:**
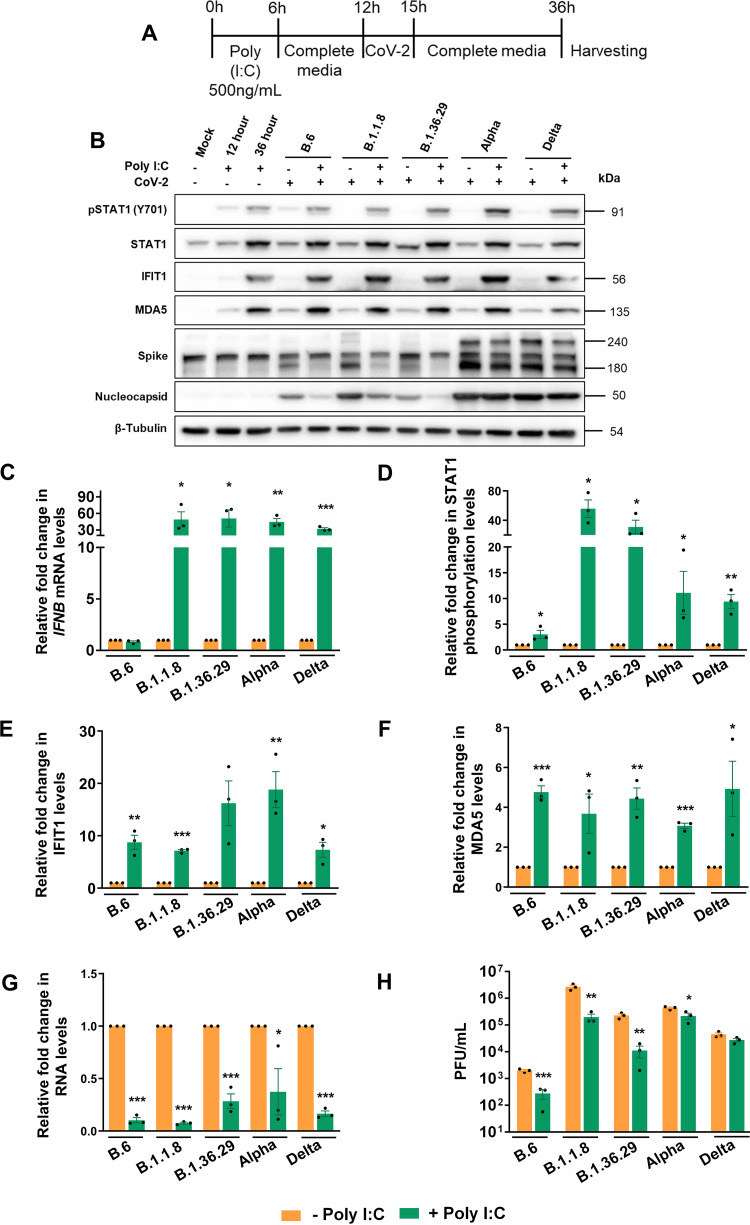
Alpha and Delta are sensitive to RLR activation by poly(I·C). (A) Schematic of the experimental setup for poly(I·C) treatment prior to variant infections. Caco2 cells were transfected with 500 ng/mL poly(I·C) for 6 h, after which the transfection medium was replaced with growth medium for incubation for another 6 h. At this point, the cultures were infected with 1 MOI of individual variants with 3 h of inoculation followed by further incubation in virus-free medium for a total of 24 h infection. (B) Immunoblots of the samples prepared from the infection for analyzing JAK-STAT activation. (C) *IFNB1* quantification in poly(I·C)-treated infected samples against the untreated infected samples by qRT-PCR. The values are represented as fold changes. First, fold changes from the infected samples against the mock-infected samples were generated through the ΔΔ*CT* method by normalizing against the internal control GAPDH of the corresponding sample. Subsequently, fold changes of such values generated in the poly(I·C)-treated infected samples against the untreated samples were calculated and plotted in the graph. (D to F) Densitometric quantification of the STAT1 phosphorylation and expressions of IFIT1 and MDA5. (G) SARS-CoV-2 RNA levels in the supernatants of poly(I·C)-treated infected samples measured by qRT-PCR and represented as fold changes against the values from the respective untreated infected samples. First, fold changes from the infected samples against the mock-infected samples were generated through the ΔΔ*CT* method by normalizing against the internal control RNase P of the corresponding sample. Subsequently, the fold changes of such values generated in the poly(I·C)-treated infected samples against the untreated samples were calculated and plotted in the graph. (H) Infectious titers of SARS-CoV-2 in the supernatant of poly(I·C)-treated infected samples measured by PFA. Raw values were plotted as PFU/mL for specific time intervals and individual variants. All the graphs are representatives of biological triplicates. A common color legend describing the variant identity has been provided at the bottom of the figure. All graphs were prepared using GraphPad Prism version 8.0.2. Statistical significance is represented as *, **, and *** for *P* < 0.05, *P* < 0.01, and *P* < 0.005, respectively.

Our results clearly demonstrated that B.6, B.1.1.8, and Alpha have progressively developed capabilities to delay RLR and IFN signaling pathways, whereas Delta and B.1.36.29 have further evolved to silence the responses throughout the infection time course. We asked if Delta could evade the prior activation of the RLR pathway where the previously activated RLR pathway would be suppressed by its infection. Caco2 cells transfected with poly(I·C) for 12 h were infected with the variants for 24 h (Fig. 3A). While the induction of *IFNB1* confirmed the activation of RLR following poly(I·C) (Fig. 3C) treatment, STAT1 phosphorylation (Fig. 3B and D) accompanied by elevated IFIT1 and MDA5 levels (Fig. 3B, E, and F) indicated the activation of JAK-STAT pathways. Though poly(I·C) augmented STAT1 phosphorylation in B.6 infection, its extent was masked by the higher basal level of phosphorylation caused by the infection (Fig. 3B and D). A similar masking was also seen in *IFNB1* levels in B.6 infection (Fig. 3C) that caused robust *IFNB1* activation at 24 hpi (Fig. 2B). The treatment resulted in an appreciable drop in N levels in B.6, B.1.1.8, and B.1.36.29 infections, but not in Alpha and Delta variants (Fig. 3B). Poly(I·C) inhibited RNA replication of all variants (Fig. 3G), indicating that genomic RNA replication of all SARS-CoV-2 variants is susceptible to the prior activation of the RLR pathway. However, poly(I·C) had only a partial impact on the infectious titers of Alpha and Delta, while the other variants were susceptible (Fig. 3H). These results clearly indicated that early activation of the RLR pathway prior to infection is efficient enough to restrict SARS-CoV-2, but the later variants are able to partially overcome this restriction.

**FIG 4:**
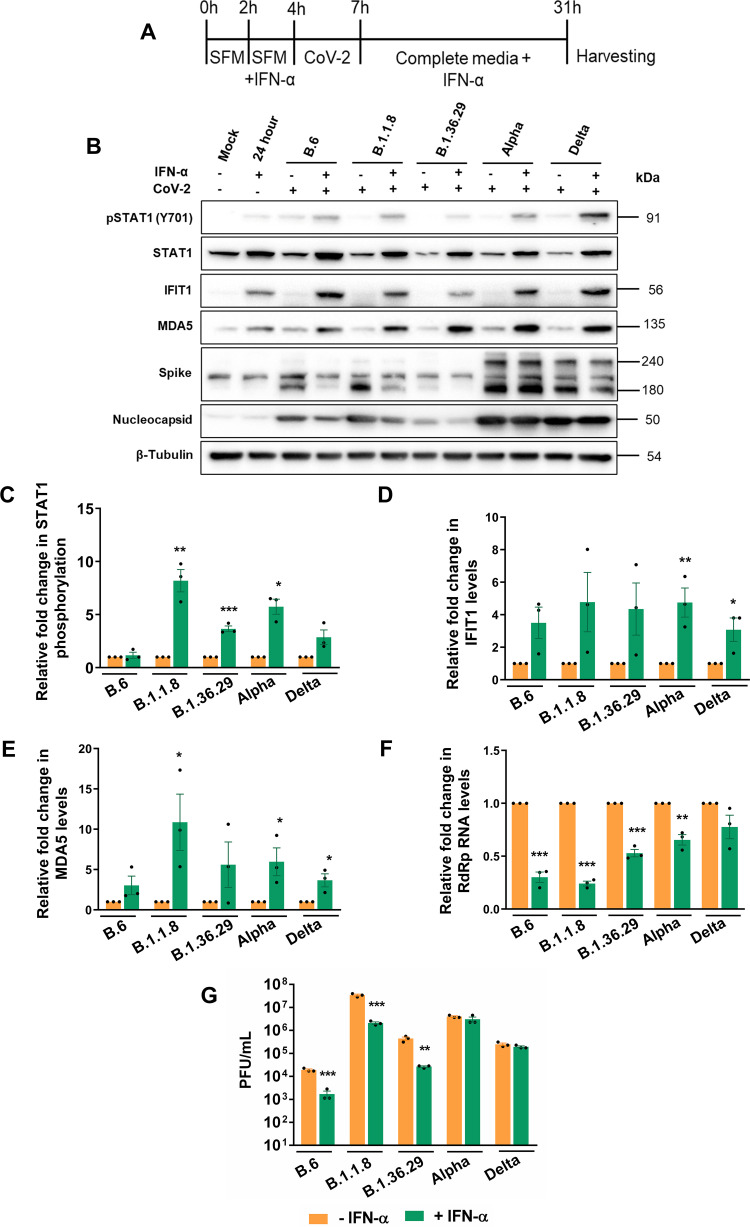
Alpha and Delta show resistance to IFN. (A) Schematic of the experimental setup for IFN-α treatment prior to variant infections. Two hours prior to IFN-α treatment, Caco2 cells were incubated with SFD. IFN-α containing SFD was added to the cells at a concentration of 500 U/mL of IFN-α, and further incubated for 4 h. Cells were infected in SFD in the absence of IFN-α for 3 h, after which the inoculum was replaced with growth medium containing IFN-α and incubated until 24 hpi. (B) Analysis of the JAK-STAT pathway activation following IFN-α treatment by immunoblotting STAT1 phosphorylation and expression of IFIT1 and MDA5. (C to E) Measurement of STAT1 phosphorylation and expression of IFIT1 and MDA5 by densitometry. (F) Measurement of SARS-CoV-2 RNA levels in the supernatants of IFN-α-treated infected samples by qRT-PCR, which is represented as fold changes against the values from the respective untreated infected samples. As in Fig. 3G, fold changes from the infected samples compared to the mock-infected samples were generated first through the ΔΔ*CT* method by normalizing against the internal control RNase P of the corresponding sample. Subsequently, fold changes of such values generated in the poly(I·C)-treated infected samples compared to the untreated samples were calculated and plotted in the graph. (G) Infectious titers of SARS-CoV-2 in the supernatant of IFN-α-treated infected samples measured by PFA. Raw values were plotted as PFU/mL for specific time intervals and individual variants. A common color legend describing the variant identity has been provided at the bottom of the figure. All graphs were prepared using GraphPad Prism version 8.0.2. Statistical significance is represented as *, **, and *** for *P* < 0.05, *P* < 0.01, and *P* < 0.005, respectively.

We then studied the sensitivity of SARS-CoV-2 variants to type I IFN. Though SARS-CoV-2 proteins are shown to intercept STAT1 phosphorylation, leading to its inactivation, IFNs are also shown to restrict SARS-CoV-2 replication (23, 27). IFN-α treatment of Caco2 cells (Fig. 4A) activated the JAK-STAT pathway, which is evident from increased STAT1 phosphorylation (Fig. 4B and C) and elevated levels of IFIT1 and MDA5 (Fig. 4B, D, and E). The treatment brought about a considerable reduction in N levels in B.6, B.1.1.8, and B.1.36.29, but not in Alpha and Delta infections (Fig. 4B). IFN-α treatment caused a significant drop in viral RNA titers in B.6, B.1.1.8, and B.1.36.29, but much less for Alpha and Delta infections, with Delta displaying the highest resistance (Fig. 4F). Infectious titers of B.6, B.1.1.8, and B.1.36.29 were also significantly lower upon IFN-α treatment, but not much for Alpha and Delta (Fig. 4G), indicating that the latter two variants have acquired resistance to IFN-α signaling but are susceptible to RLR pathway activation. These results also suggest that the poly(I·C)-mediated restriction of SARS-CoV-2 is less dependent on IFN pathways but uses noncanonical mechanisms against which SARS-CoV-2 has not gained resistance. Collectively, our results indicated a gradual and independent evolution of mechanisms to resist IFN-dependent and -independent antiviral mechanisms by the recent SARS-CoV-2 variants.

### Gene expression profiling reveals strong inactivation of antiviral pathways by Delta.

**FIG 5:**
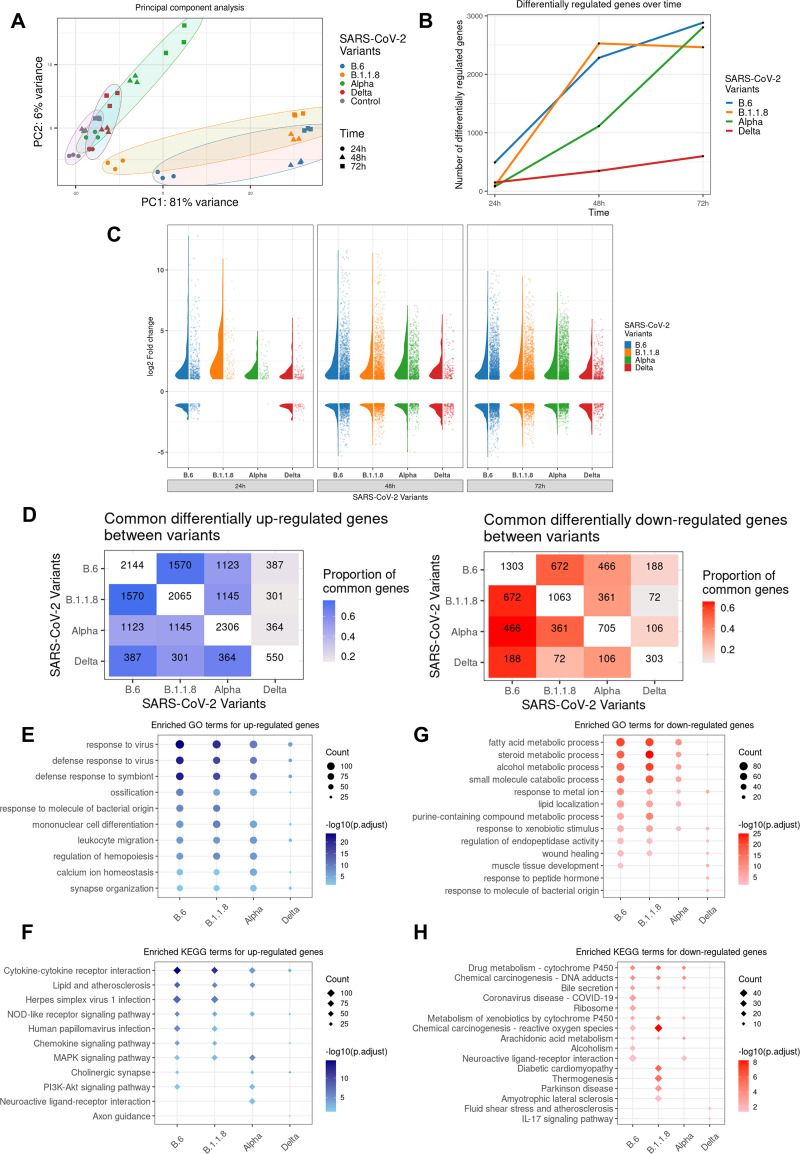
Gene expression profiling in response to SARS-CoV-2 variants. Total RNA isolated from Caco2 cultures infected with distinct variants for the respective time intervals was subjected to next-generation sequencing. Three biological replicates were used for library generation and sequencing. The sequences generated were analyzed by PCA. All DEGs considered had log_2_ fold changes of >1 for the upregulated and <–1 for the downregulated genes, with a *P* adjacent value of <0.05. (A) PCA analysis of the sequences generated. Regularized log transformed count data were used for computing principal components. The PCA confirms the quality of data where biological replicates clustered together. From the control samples, maximum variance was observed for B.6 and B.1.1.8, followed by Alpha. (B) Line graphs showing the total number of differentially expressed genes in response to individual variants at the specified time points. The number represents sum of both up- and downregulated genes. (C) Violin plots representing the distribution of log_2_ fold changes and jitter plots representing the number of DEGs in response to different variants at each time point. (D) Heat maps representing the overlapping DEGs across time points between variant-infected samples in the *x* axis and *y* axis. The numbers in diagonal boxes represent the total number of statistically significant up- or downregulated genes in the corresponding samples. The upregulated genes are represented in blue boxes, while the downregulated ones are in red. The color intensity represents the proportion of DEGs for the variants in the *y* axis overlapping DEGs for the variants in the *x* axis. (E and F) Enrichment analysis representing the enriched GO (circles) and KEGG (diamond) terms for upregulated DEGs for each variant-infected sample across time points. The size of the dot is proportionate to the number of DEGs representing the enriched term, and the intensity of the color represents the -log_10_ (adjusted *P* value) of the DEGs represented. (G and H) Similar enrichment analysis for downregulated genes caused by infection by individual variants. The upregulated DEGs are represented in blue, while the downregulated ones are in red.

We analyzed the time course transcriptional reprograming (TR) following infections by individual variants of SARS-CoV-2 except B.1.36.29 in Caco2 cultures. B.1.36.29 infection was not included, as this variant lacked the advanced feature of IFN resistance and was not a prominent variant in circulation. Principal-component analysis (PCA) confirmed that the biological replicates clustered together, and maximum variance was observed for B.6 and B.1.1.8, followed by Alpha compared to the controls, while Delta showed the least variations (Fig. 5A). This suggests a strong host transcriptional response to B.6, B.1.1.8, and Alpha, but not to Delta. The number of differentially expressed genes (DEGs) suggests that B.6 caused the sharpest response, followed by B.1.1.8 and Alpha in that order (Fig. 5B). Alpha caused a comparable scale of TR at 72 hpi but was significantly delayed compared to B.6 and B.1.1.8, indicating a better control of host response by this variant. IRF3 phosphorylation remained muted, and *IFNB1* and *IFNL1* levels were uninduced even at 72 hpi by Alpha (Fig. 2A to C). Unlike B.6, B.1.1.8, and Alpha infections, Delta caused steady, benign TR throughout the time course (Fig. 5B). Interestingly, the overall distribution of DEG fold changes by Alpha remained much lower than those from B.6 and B.1.1.8 (Fig. 5C). The highest distribution for B.6 and B.1.1.8 was found at 48 hpi, while for Alpha, it was seen at 72 hpi. Unlike in the case of other variants, the distribution of DEG fold changes was maintained throughout the time course in Delta infection, suggesting that it is able to tightly suppress the host response. The TR imprint of Alpha more closely resembled those of B.6 and B.1.1.8 than that of Delta, while that of Delta overlapped closely with both B.6 and Alpha (Fig. 5D). Gene Ontology (GO) analysis of the consolidated DEGs demonstrated a strong enrichment of genes participating in antiviral response for the upregulated genes in B.6, B.1.18, and Alpha infections, but not in Delta (Fig. 5E). Further, mononuclear differentiation and leukocyte migration factors were strongly enriched in B.6, B.1.18, and Alpha infections compared to Delta, indicating that Delta infection does not alarm the adaptive immune response (Fig. 5E), particularly from 48 hpi (Fig. S2A). Stronger enrichment of DEGs from 48 hpi underlined the delayed response to SARS-CoV-2. KEGG analysis identified substantially reduced enrichment of genes involved in cytokine-chemokine, NF-κB, tumor necrosis factor (TNF), nod-like receptor (NLR), and phosphatidylinositol 3-kinase (PI3K)-AK strain transforming protein (AKT) signaling pathways in Delta infection compared with B.6, B.1.1.8, and Alpha infections (Fig. 5F, Fig. S2B). Among the downregulated genes in B.6, B.1.1.8, and Alpha infections, enrichment was found for processes involved in fatty acid metabolism and lipid localization, particularly beyond 48 hpi, indicating unique associations of Delta with the host-derived membranous compartment (Fig. 5G, Fig. S3A). Because membrane components are very critical for the SARS-CoV-2 life cycle, their metabolism is modulated by the viruses for their benefit. Though B.6 and Delta infections caused transcriptional downregulation of several genes at 24 hpi (Fig. 5C), no significant functional enrichment was observed for these genes from Delta samples (Fig. S3A and B). KEGG enrichment analysis showed a Delta-specific downregulation of a small set of components of proinflammatory interleukin-17 (IL-17) and TNF signaling pathways and cytokine-cytokine interaction, late in infection (Fig. 5H, Fig. S3B), indicating that Delta not only spares cytokine induction, but inhibits it at the later stages of infection. The progressively depleted proportion of the regulated genes B.6 shares with B.1.1.8, Alpha, and Delta in that order indicated a continuing divergence of the evolving variants from the earliest variant, B.6 (Fig. S4A). Only a small fraction of DEGs across all time points overlapped the four infections to form a pool of commonly regulated genes (261 up- and 57 downregulated), indicating the unique transcription profiles generated by the individual variants (Fig. S4A and S5A and B). Among the 261 commonly upregulated genes, significant enrichment was seen for antiviral response processes in GO analyses (Fig. S4B). The lack of enrichment for genes uniquely associated with individual variants indicated that the functional significance of a significant proportion of DEGs cannot be ascertained for each of the variants (Fig. S4B and D). The commonly downregulated genes (Fig. S4D) did not form any enrichment, while those from B.6, B.1.1.8, and Delta formed individual enrichment groups (Fig. S4C and E). Delta caused the lowest magnitudes of gene activation and suppression among the common set of DEGs across the variants. (Fig. S5A and B).

### Delta infection causes more intense and persistent subversion of cytokines, chemokines, and antigen presentation genes than Alpha.

**FIG 6:**
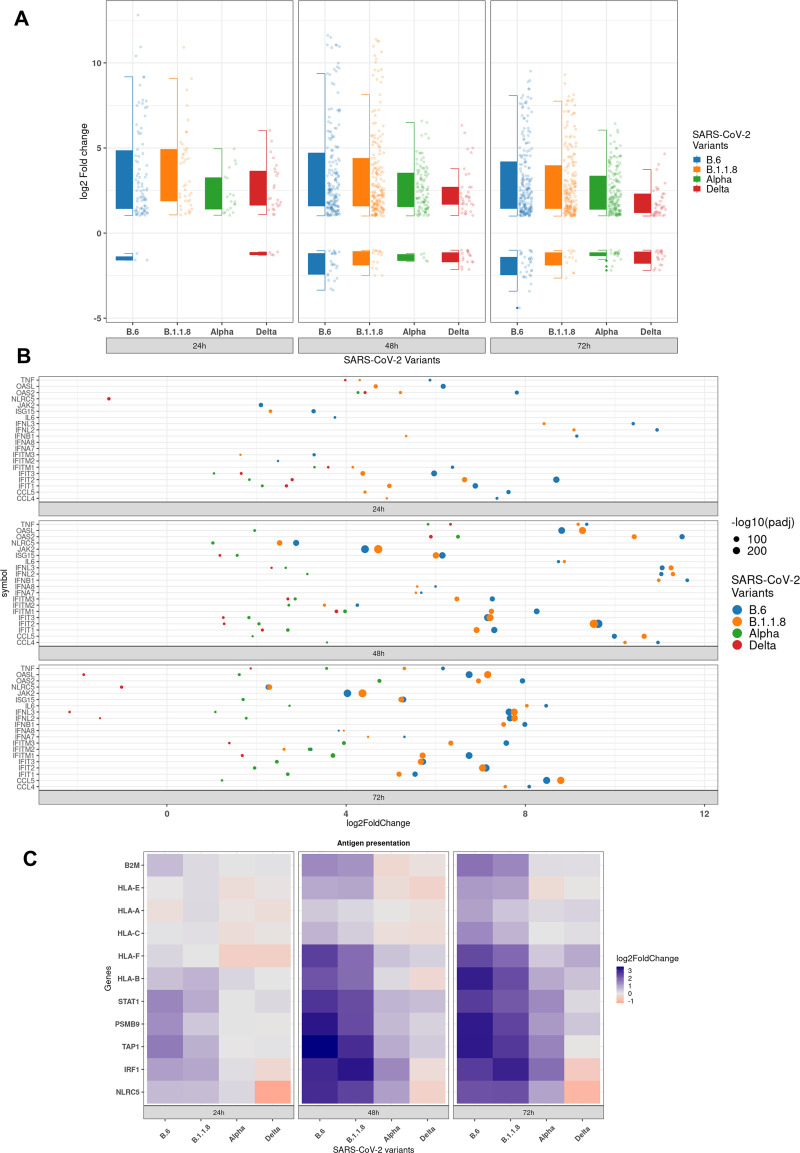
Activation of antiviral immune genes is severely suppressed in Delta infection. (A) Boxplot representing the distribution of log_2_ fold change and dot plot representing the number of differentially expressed genes (DEGs, 823) annotated for antiviral functions for specified time points. (B) Jitter plot representing the log_2_ fold change of 20 select genes participating in the innate immune response. The size of the dot represents the -log_10_ adjusted *P* value. (C) Heat maps representing the log_2_ fold change of genes participating in antigen presentation in response to the individual variants at the specified time points.

Delta not only caused a low-grade TR of antiviral genes, but lower quantum of differential expression as well (Fig. 6A and B), maintaining a steady profile with no major changes during the time course, further suggesting that it has developed capabilities to persistently silence the response. The box plot considered 822 genes classified under various processes contributing to innate immune response, response to cytokine, defense response, type-I IFN pathway, and leukocyte activation and differentiation. In line with our earlier data, only a small set of genes were reprogrammed by Delta infection (Fig. 6A). Within a select subset of these genes, Delta specifically downregulated several antiviral genes of interest, such as *OASL*, *NLRC5*, *IFNL2*, and *IFNL3*, at 72 hpi (Fig. 6B). Downregulated genes in Delta also enriched for cytokine receptor interaction and TNF and IL-17 signaling (Fig. 6B and Fig. S3B), indicating the distinct influence of this variant on the host response. Alpha and Delta suppressed type I IFN induction, whereas B.6 and B.1.1.8 induced *IFNB1* from 24 hpi. Type III IFNs, the early-responding cytokines in epithelial cells, were detected early in B.6 and B.1.1.8 infections and later in Alpha infection (*IFNL1*, *IFNL2*, and *IFNL3*) at 48 hpi, thereby indicating that the late surge of TR in Alpha infection could partly be triggered by this class of IFNs (*IFNL2* and *IFNL3* in Fig. 6B; *IFNL1* in Fig. S6). Consistent with these observations, ISG activation was also very limited in Delta infection (Fig. S6 and 7A). RLR and NLR pathway components were also significantly activated by B.6 and B.1.1.8, and to a moderate level by Alpha (Fig. S7B and 8A, respectively). The absence of any appreciable activation of NF-κB by Delta compared with the others was in agreement with the earlier observations (Fig. S8B). Intriguingly, despite a clear absence of both type I and III IFNs, a limited set of ISGs (*OAS2* and a few *IFIT*s) were activated by Delta, indicating the activation IFN-independent pathways (Fig. 6B and Fig. S6). Proinflammatory chemokines *CCL4* and *IL-6*, which promote the cytokine storm, were activated only by B6 and B.1.1.8 (Fig. 6B and Fig. S7 and S9). However, TNF-α expression was detected in Delta infection (Fig. 6B and Fig. S8B and S9C), albeit later and milder than the previous variants, indicating that its regulation is independent from that of *CCL4* and *IL-6*. These data, agreeing with the immunoblot data (Fig. 1 and 2), confirm that the recently emerged variants have evolved mechanisms to suppress both type I and III IFN, as well as cytokine and chemokine activations.

Additionally, antigen presentation was also compromised in Delta infection. A study had previously reported the inhibition of activation of major histocompatibility complex (MHC) class-I pathways by SARS-CoV-2, where they analyzed the results until 24 hpi (28). We detected similar results but found their activation at later hours of infection. While the regulators *NLRC5*, *IRF1*, *STAT1*, and *HLA-B* were progressively activated through the infection time course in B.6 and B.1.1.8 infections, they were hardly detected in Delta infection (Fig. 6C and Fig. S6E and S9). Collectively, our results demonstrate that Delta infection causes a very mild response from the host cells, thereby possibly resulting in a delayed or milder activation of the adaptive immune response.

## DISCUSSION

**FIG 7:**
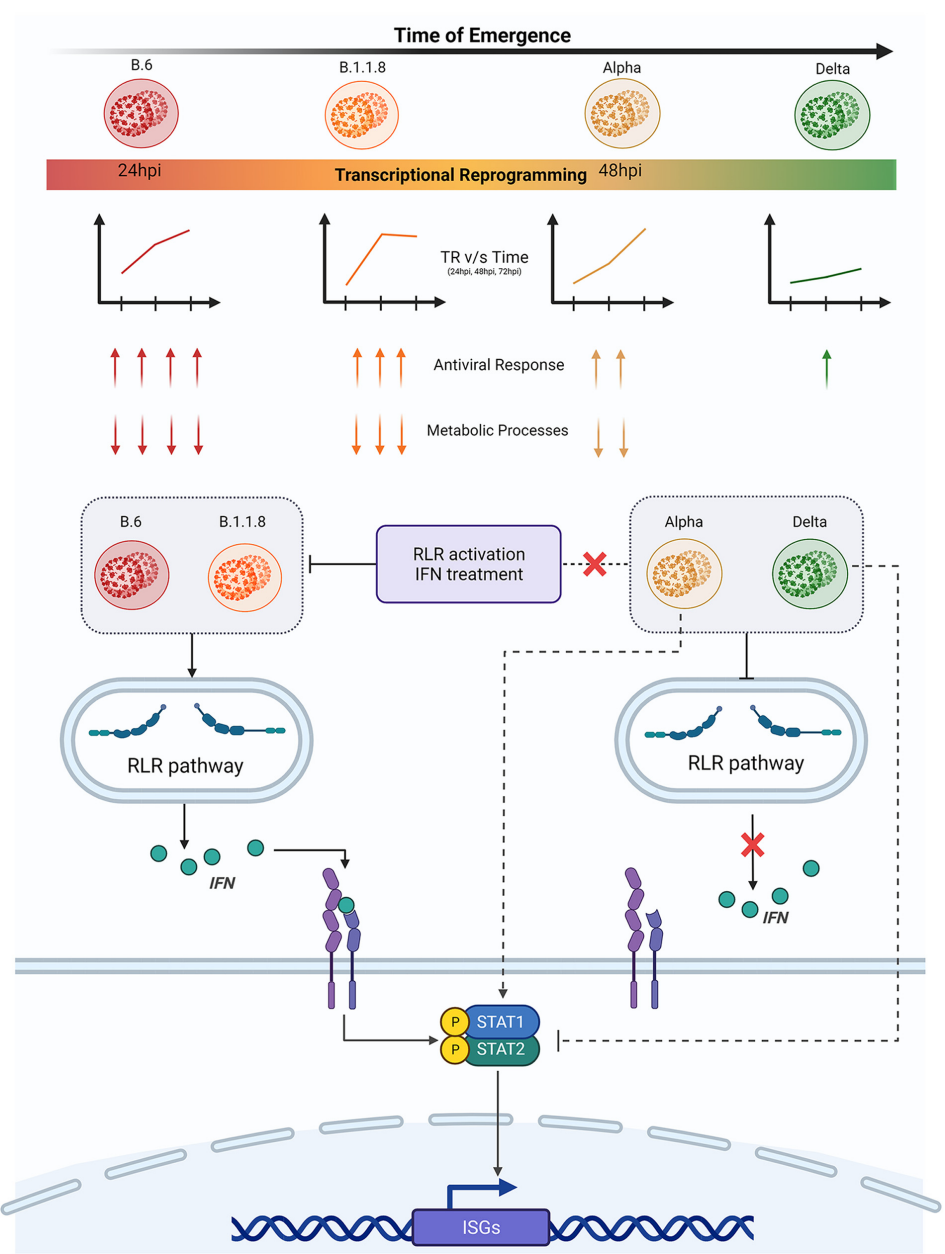
The Delta variant has gained highly advanced control over the innate immune response and suppresses host responses effectively. The variants that emerged during the early part of the COVID-19 pandemic trigger a moderate immune response by 24 h and a robust response by 48 h postinfection. This was evident by the activation of the RLR pathway, which was further substantiated by transcriptome data. However, Alpha suppresses the RLR pathway effectively but failed to suppress STAT1 phosphorylation, possibly through an IFN-independent mechanism. This was reflected in the late surge of transcriptional activities in Alpha infection. Delta has been the most advanced in suppressing not just the innate immune response, but the host response in general. Delta suppressed the RLR pathway, IFN production, and STAT1 phosphorylation, and this was reflected in the modest, steady response from the infected cells throughout the infection period. SARS-CoV-2 variants used in this study are presented from left to right based on their time of emergence, with B.6 being the earliest and Delta being the most recent. The color of the variant virus particle shown in the schematic directly correlates with the degree of transcriptional reprogramming by variants presented in the graphical depiction below individual variants. The color intensity of the rectangular bar represents transcriptional reprogramming and control over the host immune response by individual variants. Red represents elevated TR and strong activation of immune response, while green represents minimal TR and greater control over host responses. Two of the GO enriched terms are shown with arrows. The numbers of arrows represent the potency of activation or inhibition, where up arrows indicate upregulation of DEGs involved and down arrows indicate downregulation. The variants studied here broadly fall under two groups based on the regulation of RLR pathway components and their response to activated innate immune responses [RLR activation by poly(I:C) and JAK-STAT activation by IFN treatment]. B.6 and B.1.1.8 activated RLR signaling followed by IFN secretion and ISG expression via the JAK-STAT axis. RLR and JAK-STAT signaling remain suppressed in Delta infection. Uniquely, Alpha follows the noncanonical mode of STAT activation without any detectable expression of IFNs.

Viral infections are studied from the perspective of virulence and transmissibility, which often share diffused borders. Recent developments in the sequence determination of variants have given better insight into the process of viral and virulence evolution (11). Traditional wisdom suggests that the virulence caused by a pathogen in a new host would be tempered over a period of their coexistence driven by natural selection. Though a trade-off between the virulence and transmission rate is often observed during the evolution of the relationship with the host, it may not be necessary (11, 29). In this study, we attempted to comprehensively characterize how the new variants that emerged during the pandemic have evolved with the host from the point of the host response to these individual variants. Our study clearly demonstrates a spectrum of host responses triggered by distinct viral variants, where the earliest one, B.6, caused the quickest, while the latest one, Delta, caused the most benign response, suggesting that these variants have been able to effectively contain multiple surveillance mechanisms of the host and thus have a stricter control over host responses. The responses against the other two variants were indications of the measured progression of the virus to a more benign variant (Fig. 7). The variants that emerged later have evolved better mechanisms to delay and to silence the innate immune response than those of the previous variants, facilitating their longer stay in the infected host. This trend of progressive delay in the host responses to B.1.1.8 and Alpha and the mild response to Delta indicated that the recently emerged variants have better mechanisms to evade the host surveillance than their earlier variants. By this criterion, they can be identified as more evolved. This trait is likely to improve with the newer dominant variants emerging after Delta, such as Omicron. It is evident that suppression of innate immunity and resistance to IFN were achieved through distinct mechanisms. Our findings have important implications for the therapeutic approaches involving IFN therapy against the emerging variants.

A recent report on the evolution of Alpha to evade innate immune response more efficiently than its previous variants (24) was also captured in our studies, as we did detect substantially elevated levels of N proteins in Alpha infections. The absence of overlapping mutations shared by Alpha and Delta (Fig. S10) indicates divergent mechanisms adopted by these variants to achieve similar outcomes. Clearly, they must have targeted the innate sensing pathways RLR and TLR uniquely. A much delayed, but strong, host antiviral response against Alpha despite the suppression of the RLR pathway and *IFNB1* production indicated the involvement of alternate mechanisms that Delta was able to successfully suppress IFN-independent STAT1 phosphorylation (Fig. 2A and 7). A delayed response to Alpha also suggests that Alpha has evolved to delay the innate immune response, but probably not to evade it totally. Minimal activation of *ISG15* by Alpha and Delta indicated that its suppression might be assisting these variants in lowering the ISGylation of its target molecules that are important mediators of the innate immune response (Fig. S9G). Interestingly, despite a complete absence of IRF3 phosphorylation during Alpha infection, the host response exploded between 24 and 72 hpi, suggesting that this response is not orchestrated by IFNs.

Our studies also create a platform for further discussions on the larger question of the features that make a particular variant more transmissible. Though Delta RNA replication was the fastest, in agreement with the existing literature (9), its infectious titers were lower than those of the previous variants, an indication that silencing the host response does not appear to provide it any particular advantage in terms of its viral load. With a reported higher R_0_ for Delta (30), there is a lack of credible clinical data on its relative virulence compared with the previous variants in immunologically naive populations. Delta indeed caused severe pathology during its emerging period, while the majority of the population was unvaccinated. Currently, the possibility of conducting unbiased population studies on its severity is highly improbable due to the unavailability of immunologically naive cohorts, as would be the case of Omicron and future variants (31). The unusually high infectious titers observed for Alpha and B1.36.29 could be attributed a combination of factors, including the cellular niche and the properties associated with the variant. Most of the reports on the infectious titers of SARS-CoV-2 are generated from Vero or Vero E6 cells using the variants isolated early during the pandemic. However, certain reports indicate titers approaching 10^8^ PFU/mL (32). We have consistently observed higher SARS-CoV-2 infectious titers of supernatants generated from Caco2 cells than from Vero cells. Considering the variable tropism SARS-CoV-2 variants display in different cell models, it appears that Alpha and B1.36.29 have exceptionally high levels of tropism in Caco2 cells. Based on our data, we could speculate that the contribution by the epithelial cells to the systemic responses could be significantly lower in persons infected by Delta than by its previous counterparts. Reduced communication from the epithelial cells would also result in a lower adaptive response, thus causing lower chances of the cytokine storm. However, Delta-specific data on the cytokine storm is lacking, but our results indicate that the magnitude of the cytokine storm in Delta infections could be much smaller than that with the earlier variants. Higher incidences of respiratory support and ICU admissions were reported in Delta-prevalent regions, indicating that the lungs could have been subject to more serious damage. The results from these studies could be of great significance in characterizing the ever-evolving nature of COVID-19.

One of the potential limitations of our study is that it primarily used Caco2 cells for studying the innate immune response. Caco2 cells are extensively used in SARS-CoV-2 research, but the results from this study need to be studied in other systems, including the animal models. Additionally, our study did not address the contribution of individual viral proteins and individual mutations in them to the evolutionary progresses that the variants have achieved. Studies covering these gaps would bring about further insights into the complex mechanisms of RLR pathways and strengthen our understanding of the host-virus relationship.

## MATERIALS AND METHODS

### Cell culture, poly(I·C) transfection, and IFN-α treatment.

Vero (CCL-81) cells were purchased from Sigma-Aldrich and cultured in complete Dulbecco’s modified Eagle’s medium (cDMEM; Gibco) containing 10% fetal bovine serum (FBS; HyClone), and 1× penicillin-streptomycin cocktail (Gibco) at 37°C and 5% CO_2_. Caco2 cells, purchased from ATCC, were grown similarly but supplemented with 20% FBS. Cells were continuously passaged at 70 to 80% confluence and were maintained under conditions of ambient temperature and humidity.

Poly(I·C) transfections were performed as described in a previous report (33). Cells were seeded to reach 80% confluence. Transfection mix containing Opti-MEM-Lipofectamine 3000 poly(I·C) was prepared according to the manufacturer’s protocol and added to cells and incubated for 6 h. Later, the transfection mix was replaced with cDMEM and further incubated for 6 h. Then, 12 h later, the transfected cells were infected with virus for 3 h and further incubated in fresh cDMEM until they were harvested for analyses.

For IFN-α treatment, cells were seeded to reach 80 to 85% confluence. Cells were supplemented with serum-free DMEM (SFD) for 2 h for serum starvation. Later, SFD was replaced with fresh SFD containing 500U/mL IFN-α for 2 h (34). Following this, the cells were infected for 3 h, as described above, and incubated further with fresh cDMEM containing either phosphate-buffered saline (PBS; vehicle) or 1,000 U/mL of IFN-α (PBL Assay Science) and incubated for 24 h. Cells were harvested and used for RNA or protein work.

### SARS-CoV-2 isolates.

The five variant isolates of SARS-CoV-2 used in this study were isolated (30) at the Centre for Cellular and Molecular Biology in the biosafety level 3 facility. Their genomes were sequenced (GISAID ID EPI_ISL_458067; virus name, hCoV-19/India/TG-CCMB-O2/2020 [B.6], EPI_ISL_458046; virus name, hCoV-19/India/TG-CCMB-L1021/2020 [B.1.1.8], GISAID ID EPI_ISL_539744; virus name, hCoV-19/India/TG-CCMB-AC511/2020 [B.1.36.29], GISAID ID EPI_ISL_1672391.2; virus name, hCoV-19/India/TG-CCMB-BB649-P1/2020 [B.1.1.7], and GISAID ID EPI_ISL_2775201; virus name, hCoV-19/India/TG-CCMB-CIA4413/2021 [Delta]). The viruses were propagated in Vero (CCL-81) cells grown in SFD.

### Virus infection, quantification, and titration.

Caco2 cells were infected at 1 MOI for 3 h in SFD, after which the inoculum was replaced with complete medium and further grown until harvesting. The supernatants collected were processed for RNA preparation using a NucleoSpin viral RNA isolation kit (Macherey-Nagel GmbH & Co. KG) and infectious titer assay (plaque formation assay [PFA]). Reverse transcription-quantitative PCR (qRT-PCR) to quantify SARS-CoV-2 RNA was performed on a Roche LightCycler 480 using an nCOV-19 reverse transcription-PCR (RT-PCR) detection kit from Q-Line Molecular. Infectious titers of the supernatants were calculated using PFA as mentioned previously (35).

### Real-time quantitative RT-PCR.

Cellular RNA samples were prepared using an MN NucleoSpin RNA kit (TaKaRa). Equal quantities of RNA were reverse transcribed using Primescript reverse transcriptase (TaKaRa) following the manufacturer’s protocol. Then, 50 ng of cDNA was used for quantification using SYBR green master mix (TaKaRa) on a LightCycler 480 instrument (Roche). Transcripts of the host origin were normalized against GAPDH (glyceraldehyde-3-phosphate dehydrogenase). Relative fold changes between the experimental and control samples (2^–ΔΔ^*^CT^*) were calculated and represented in the graphs.

### Antibodies and immunoblotting.

All primary antibodies were purchased from Cell Signaling Technologies except the anti-Spike antibody (Novus Biologicals) and the anti-nucleocapsid, anti-tubulin, and anti-GAPDH (Thermo Fisher) antibodies. Horseradish peroxidase (HRP)-conjugated secondary antibodies were purchased from Jackson ImmunoResearch. Protein pellets were lysed in an NP-40 lysis buffer as described earlier (33). Protein quantification was done using the bicinchoninic acid (BCA) method (G Biosciences). The immunoblots were developed on a Bio-Rad Chemidoc MP system using ECL reagents (Thermo Fisher and G Biosciences). Quantification was performed using ImageJ.

### Next-generation sequencing.

Library preparation was done using the MGIEasy RNA library prep set (MGI) according to the manufacturer’s instructions. In brief, 500 ng total RNA was used as starting material from which rRNA was depleted using a Ribo-Zero Plus rRNA depletion kit (Illumina). The rRNA-depleted samples were fragmented and reverse transcribed, and the second strands were synthesized. DNA was then purified using DNA clean beads provided in the kit, followed by end repair and A-tailing. Barcoding and adaptor ligation were performed, and the samples were purified. Samples were amplified using adaptor-specific primers and quantified using a Qubit dsDNA high-sensitivity kit (Thermo Scientific). Sample fragment size was determined using a 4200 Tape Station (Agilent). The samples were denatured, and single-stranded circular DNA strands were generated. Further, rolling cycle amplification was performed to generate DNA nanoballs. The samples were subsequently loaded onto the flow cells (FCL) and paired end RNA sequencing was performed for read length of 100 bases.

### Data processing and analysis.

MGI adapters and low-quality reads were removed from the raw sequencing reads using Cutadapt (36). Reads with a quality score of less than 20 and smaller than 36 bp were discarded. The processed reads were then mapped to the human genome GRCh38 using HISAT2 with default parameters (37). Uniquely aligned reads were counted using the featureCounts program of the Subread package (38). Count information was available for 60,683 genes in the gtf file, downloaded from Ensembl (39). Genes with a total of 10 read counts across all the samples were removed, resulting in 35,906 genes for further analysis. Differential gene expression analysis was performed using DESeq2 (40). Genes with an adjusted *P* value of <0.05 and absolute log_2_ fold change of >1 were considered differentially expressed. For the PCA plot and heat map, the raw read counts were rlog normalized, available with the DESeq2 package.

### Functional enrichment analysis.

Functional enrichment analysis was performed using clusterProfiler (41) for GO term and KEGG pathway enrichment. We only used the biological process for GO term enrichment analysis. Similar enriched terms were further merged using the simplify function of clusterProfiler with the similarity cutoff set to 0.7. p.adjust was used as a feature to select representative terms, and min was used to select features. Wang was used as a method to measure similarity. The top 10 GO terms and KEGG pathway-based counts of genes were plotted.

### Statistical analysis.

Statistical significance was calculated by the paired-end, two-tailed Student’s *t* test method. All experiments were conducted with a minimum three independent rounds, and averaged values are represented as a scatterplot with bar graphs (depicting individual values of independent experiments). Error bars are representations of the mean ± standard error of the mean (SEM). All graphs were prepared using GraphPad Prism version 8.0.2. Statistical significance is represented as *, **, and *** for *P* < 0.05, *P* < 0.01, and *P* < 0.005, respectively.

### Institutional biosafety.

Institutional biosafety clearance was obtained for the experiments pertaining to SARS-CoV-2.

### Institutional ethics clearance.

Institutional ethics clearance (IEC-82/2020) was obtained for the patient sample processing for virus culture.

### Data availability.

The RNA sequencing (RNA-seq) data have been deposited in the Gene Expression Omnibus (GEO) database under accession number GSE193122.
