# Supplementary material for: SARS-CoV-2 Variant Delta Potently Suppresses Innate Immune Response and Evades Interferon-Activated Antiviral Responses in Human Colon Epithelial Cells
Source: Microbiol Spectr. 2022 Sep 8;10(5):e01604-22. doi: 10.1128/spectrum.01604-22 (PMC9602719; doi:10.1128/spectrum.01604-22)
Supplement: Supplemental file 1 — Download spectrum.01604-22-s0001.pdf, PDF file, 2.4 MB [file spectrum.01604-22-s0001.pdf]

SARS-CoV-2 Variant Delta Potently Suppresses Innate Immune Response and Evades Interferon-Activated Antiviral Responses in Human Colon Epithelial Cells

Dixit Tandel[1,2], Vishal Sah[1,2], Nitesh Kumar Singh[1], Poojitha Sai Potharaju[1,2], Divya Gupta[1], Sauhard Shrivastava[1,3], Divya Tej Sowpati[1,2], and Krishnan H Harshan[1,2, €]

[1]CSIR-Centre for Cellular and Molecular Biology, Hyderabad, India 500007

[2]Academy for Scientific and Innovative Research (AcSIR), Ghaziabad, India-201002

[3]Indian Institute of Science Education and Research, Bhopal, India-462066

[€]Correspondence: hkrishnan@ccmb.res.in

Supplementary Figure 1

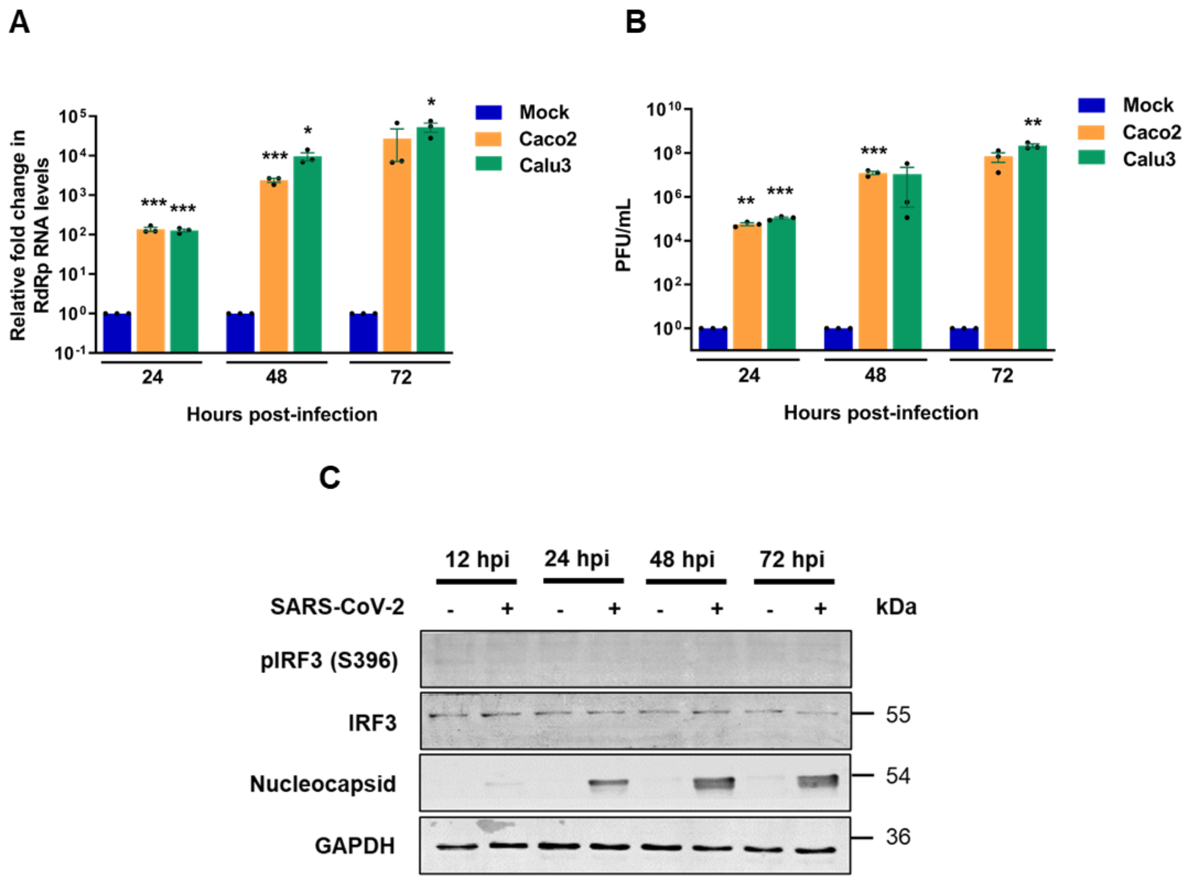

Supplementary Figure 1. Permissivity analysis for SARS-CoV-2 in Calu3 and Caco2 cells. (A) Supernatants from Calu3 and Caco2 cultures infected with 1 MOI of B.1.1.8 variant isolate of SARS-CoV-2 for 24-, 48- or 72 h were analyzed by real-time qRT-PCR to measure the genomic RNA of SARS-CoV-2. The fold-changes over the mock-infected samples were calculated by ΔΔ-Ct method and plotted in the graph. (B) Analysis of the infectious titers of SARS-CoV-2 in the supernatants generated in A, by PFA and represented in PFU/mL. (C) IRF3 phosphorylation is suppressed in SARS-CoV-2 infected Calu3 cells. Calu3 cells were infected with 1 MOI of B.1.1.8 variant isolate of SARS-CoV-2 for various time-points as indicated in the figures. Cells harvested were lyses and subjected to immunoblotting to detect viral N and phosphorylated IRF3.

Supplementary Figure 2

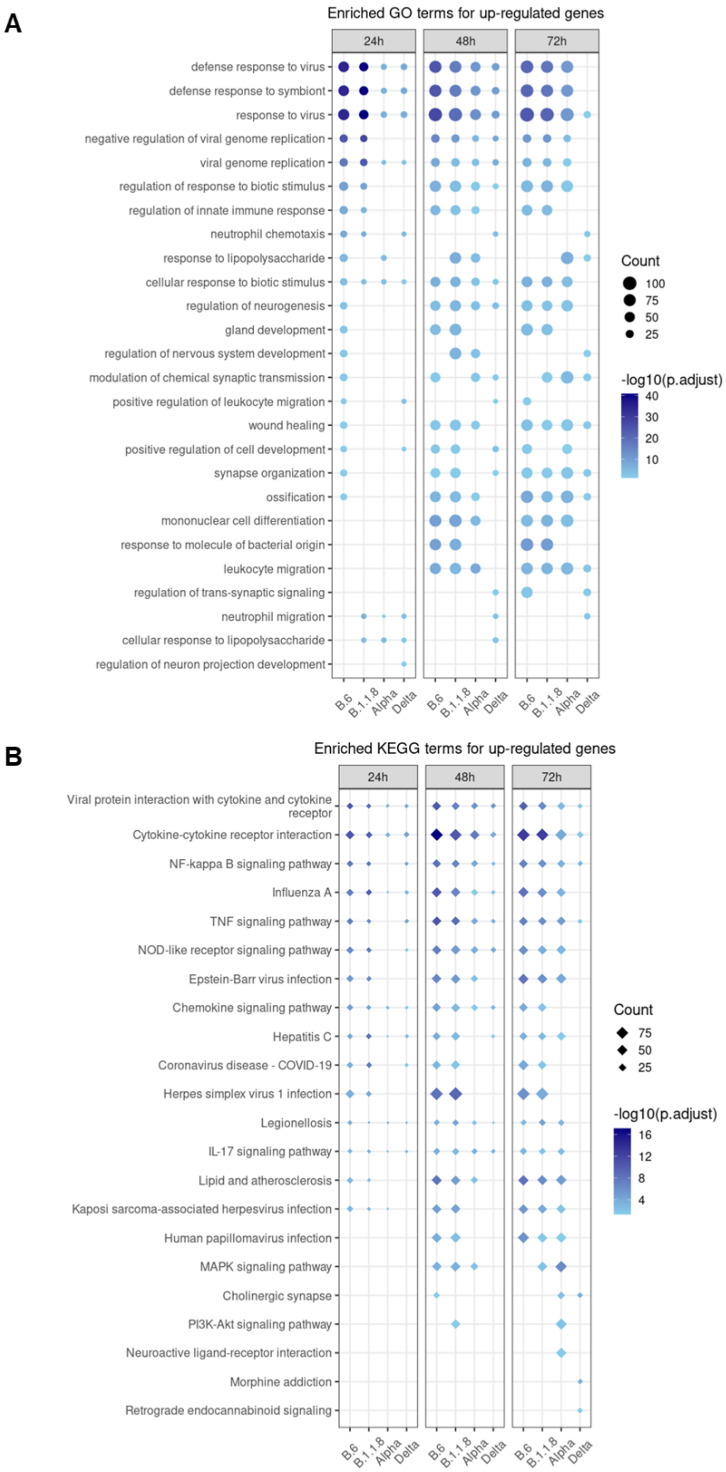

Supplementary Figure 2. Enrichment analysis representing the Enriched GO (circles) and KEGG (diamond) terms for up-regulated DEGs for each variant-infected samples at each time-points. Size of the dot represents the number of DEGs in the enriched term and the intensity of the color represents the -log10 (adjusted p-value).

Supplementary Figure 3

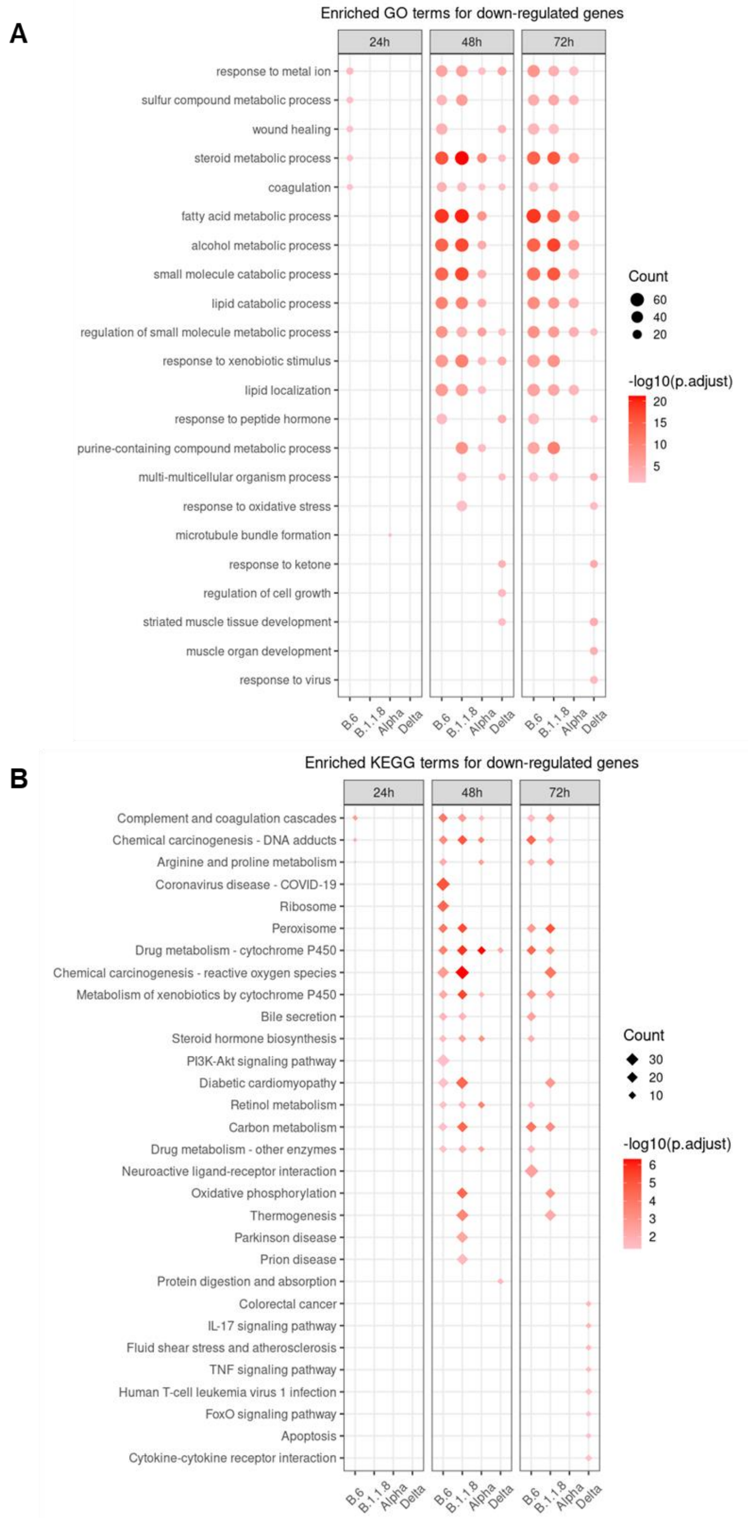

Supplementary Figure 3. (A and B) Enrichment analysis representing the Enriched GO (circles) and KEGG (diamond) terms for down-regulated DEGs for each variant-infected samples at each time-points. Size of the dot represents the number of DEGs in the enriched term and the intensity of the color represents the -log10 (adjusted p-value).

Supplementary Figure 4

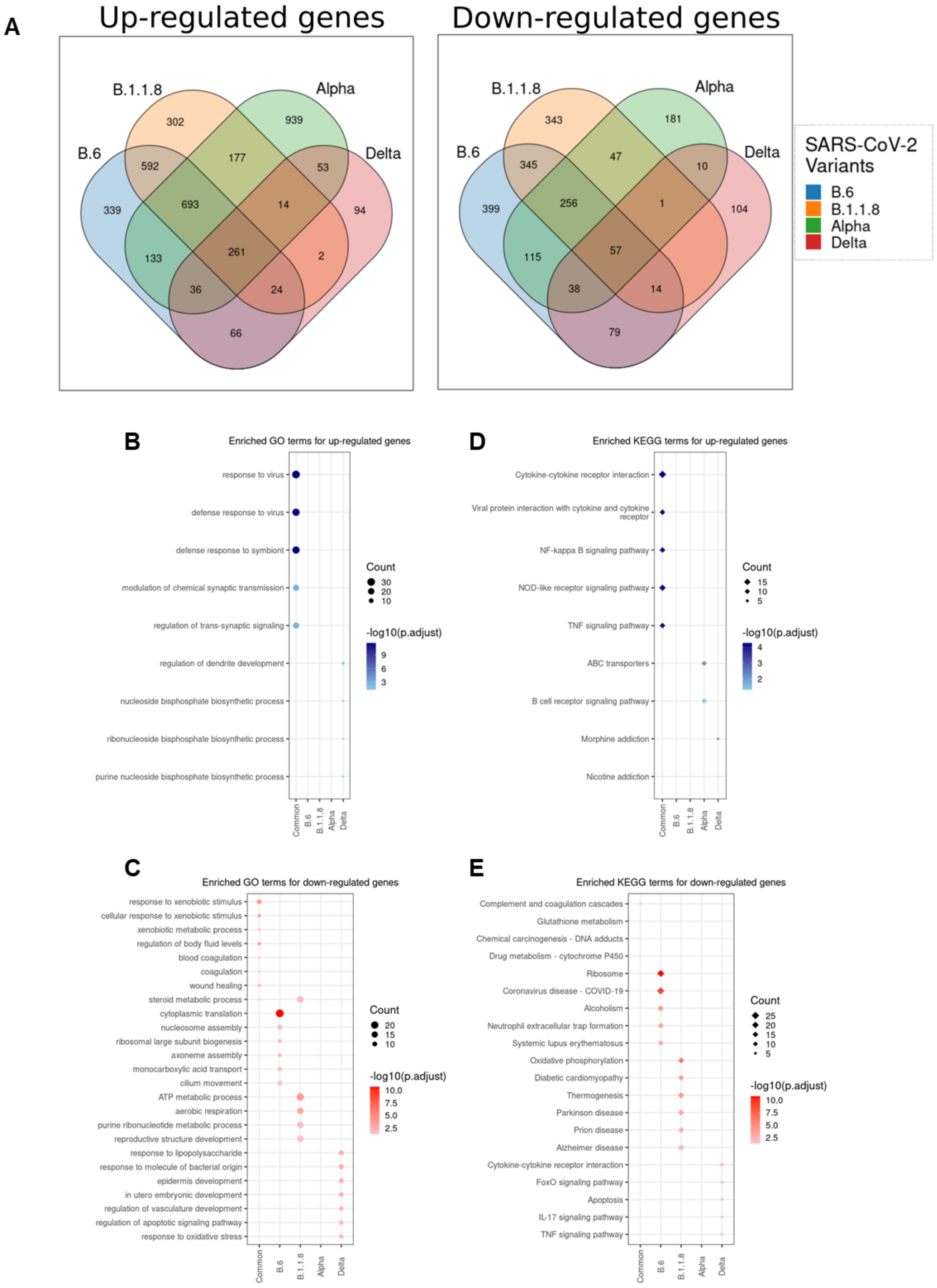

Supplementary Figure 4. Analysis of the overlapping and unique DEGs from individually infected samples. (A) Venn diagram showing the common genes that were differentially regulated by all the four variants, as well as unique genes from each individual infections, for both up-regulated and down-regulated sets. DEGs were pooled from all time-points for each variant sample and used in the analysis. (B-E) GO and KEGG enrichment analysis of the common and unique up and down regulated DEGs from each infected samples

Supplementary Figure 5

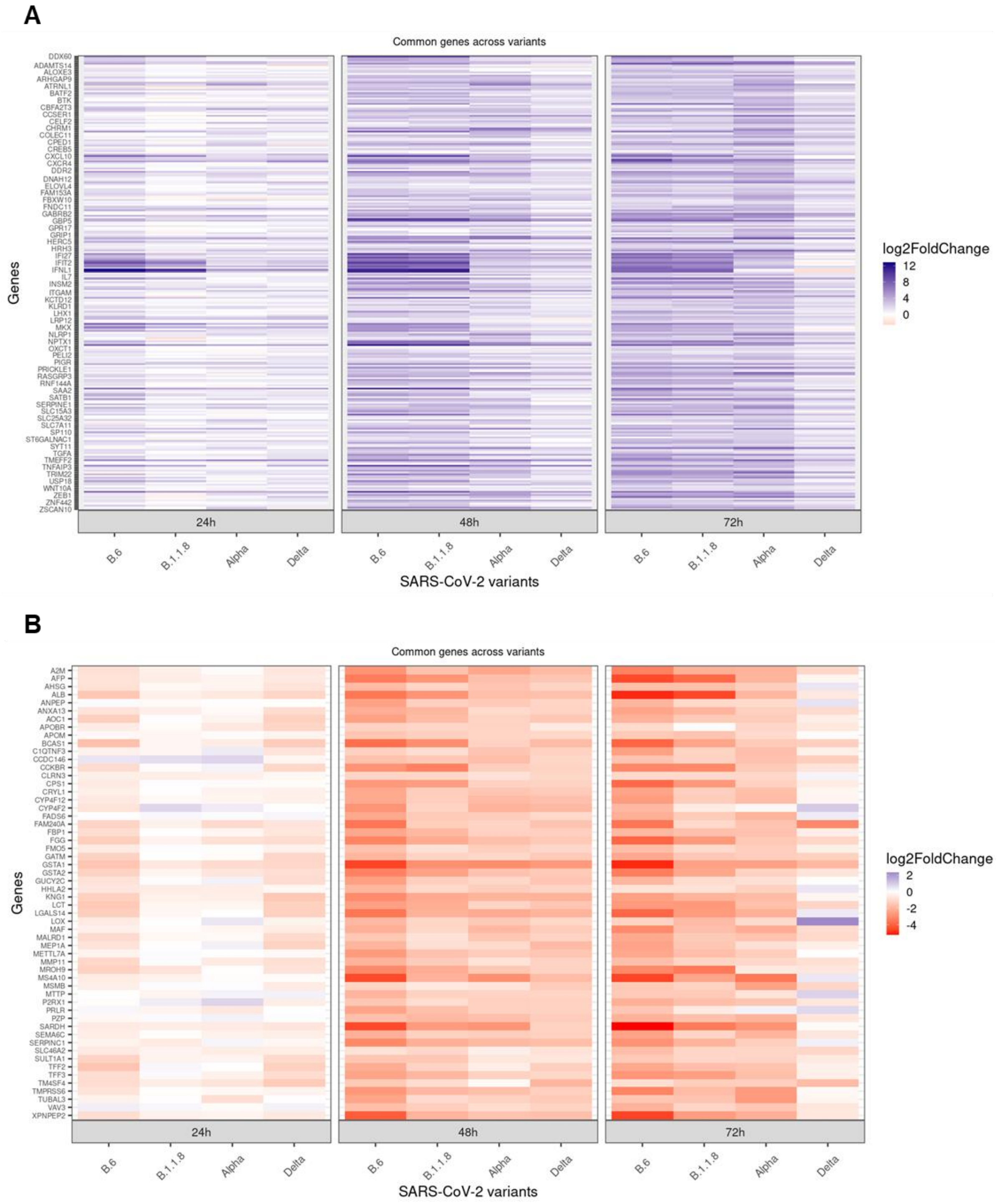

Supplementary Figure 5. Heat-maps demonstrating the log2 fold change of (A) 261 up-regulated and (B) 57 down-regulated genes, common across the four variant infections as shown in Figure 5A. The lists of genes were generated from the common pool representing DEGs from all time-points as shown in Supplementary Figure 5A.

Supplementary Figure 6

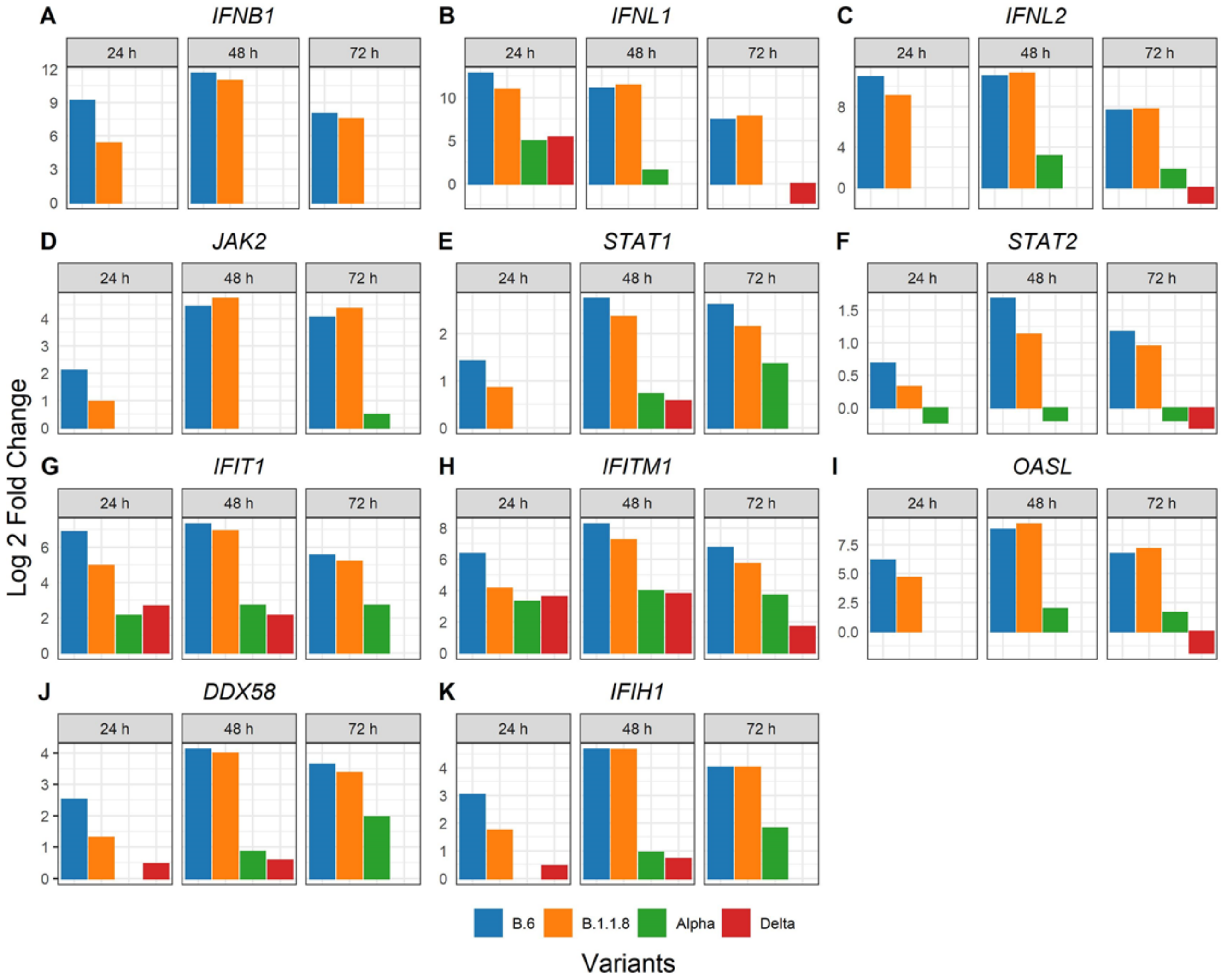

Supplementary Figure 6. Bar-graphs demonstrating the differential expression of select genes of importance from type-I and type-III IFN pathways. The graphs were generated from the *p*-value adjusted list and are statistically significant.

Supplementary Figure 7

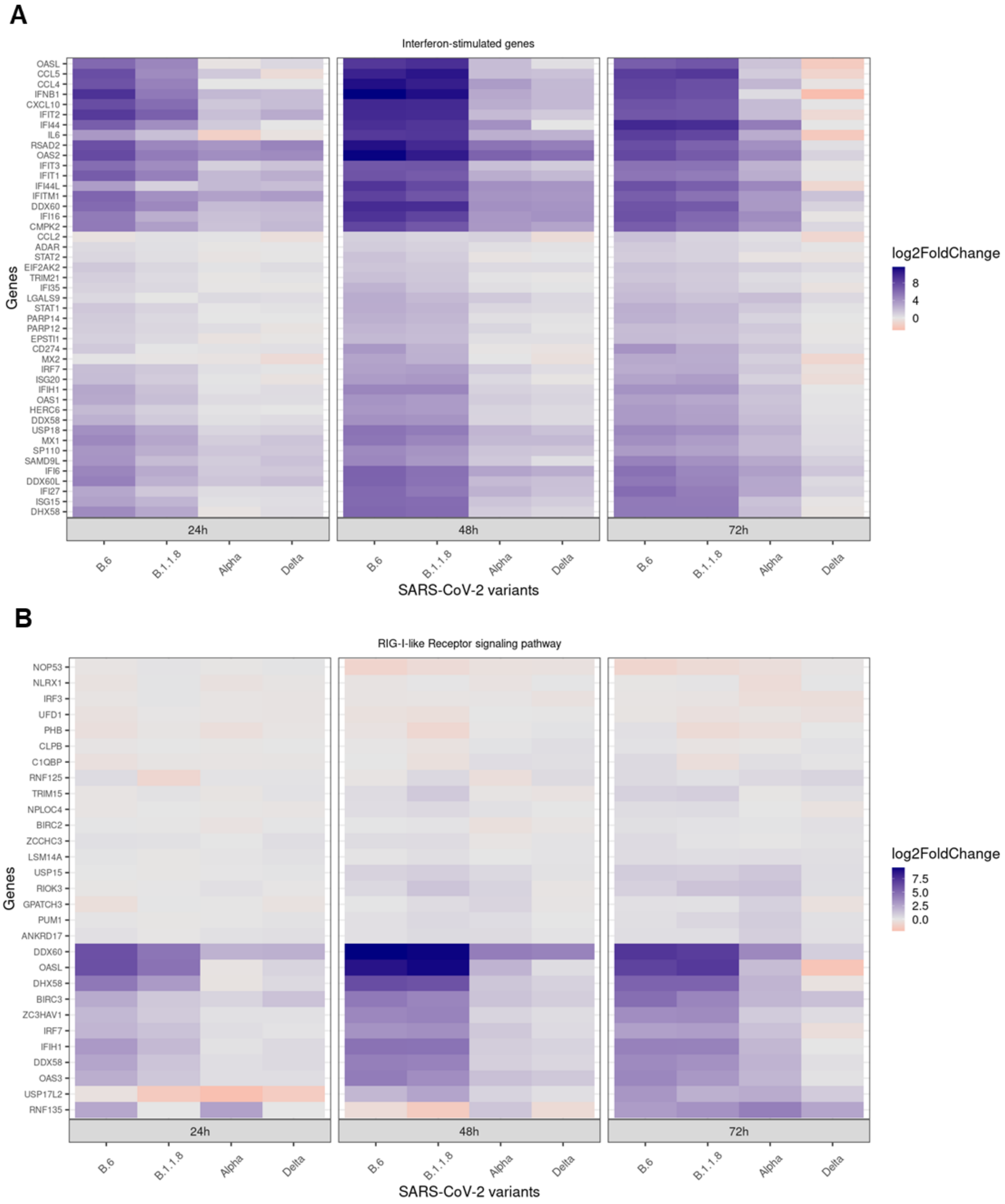

Supplementary Figure 7. (A) Heat-map demonstrating the differential expression of ISGs in response to the variant infection at specified time-intervals. (B) Heat-map demonstrating the differential expression of genes classified under RLR pathway in response to the variant infection at specified time-intervals.

Supplementary Figure 8

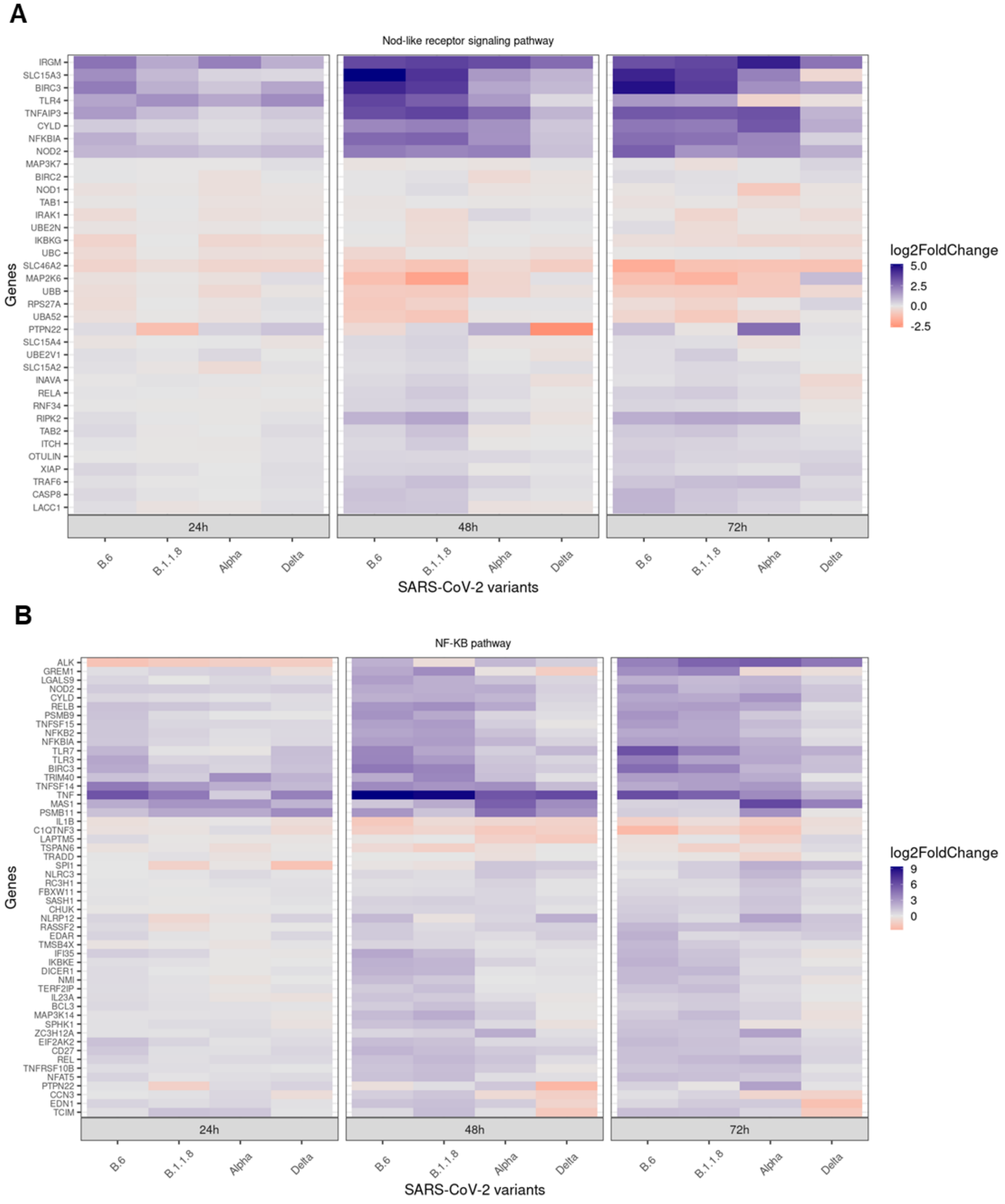

Supplementary Figure 8. Heat-map demonstrating the differential expression of genes classified under NLR pathway in response to the variant infection at specified time-intervals. (B) Heat-map demonstrating the differential expression of genes classified under NF-κB pathway in response to the variant infection at specified time-intervals.

Supplementary Figure 9

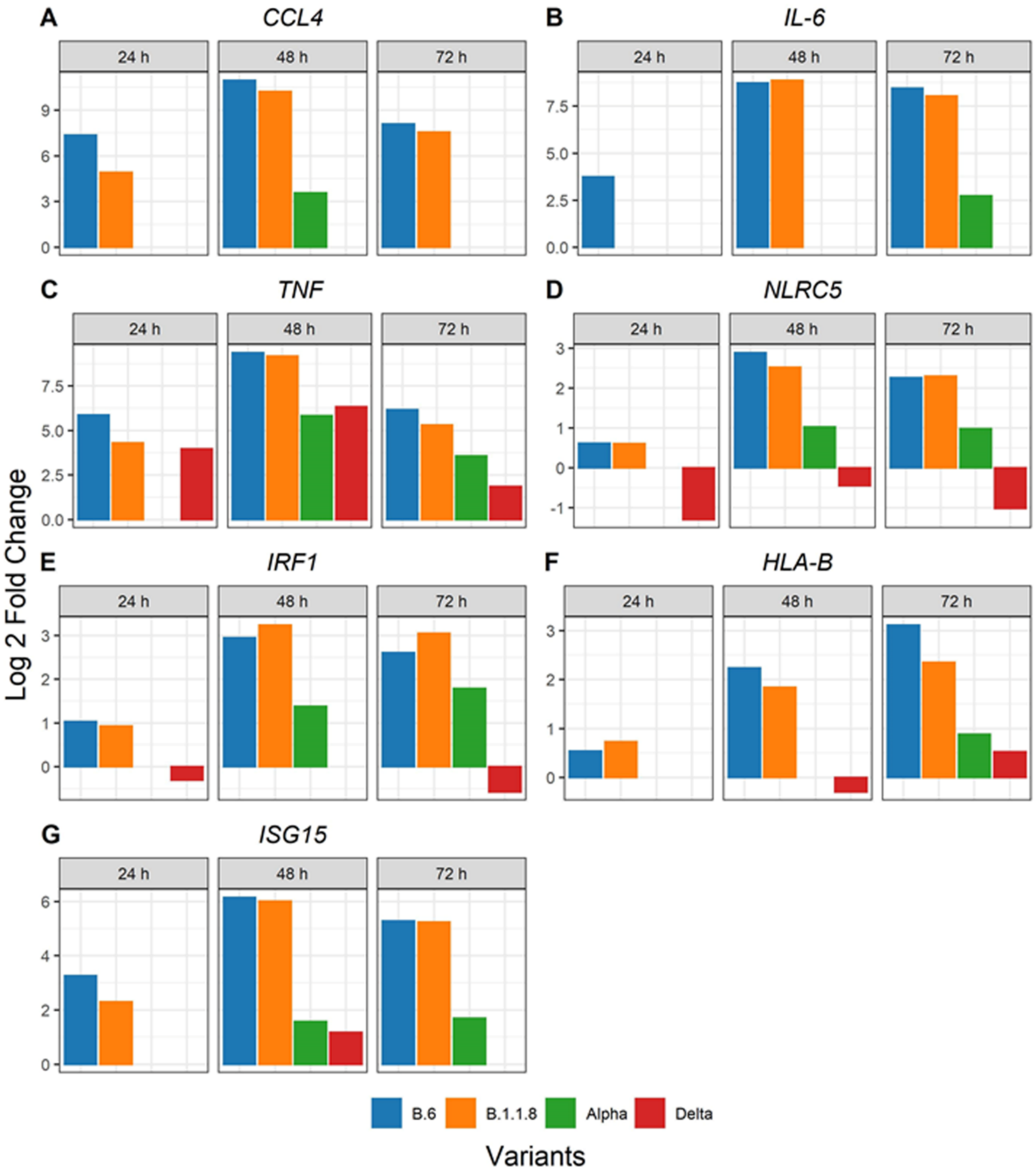

Supplementary Figure 9. Bar-graphs demonstrating the differential expression of select genes (A-F) participating in antigen presentation, and regulation of interferon pathway (G, H). The graphs were generated from the *p*-value adjusted list and are statistically significant.

Supplementary Figure 10

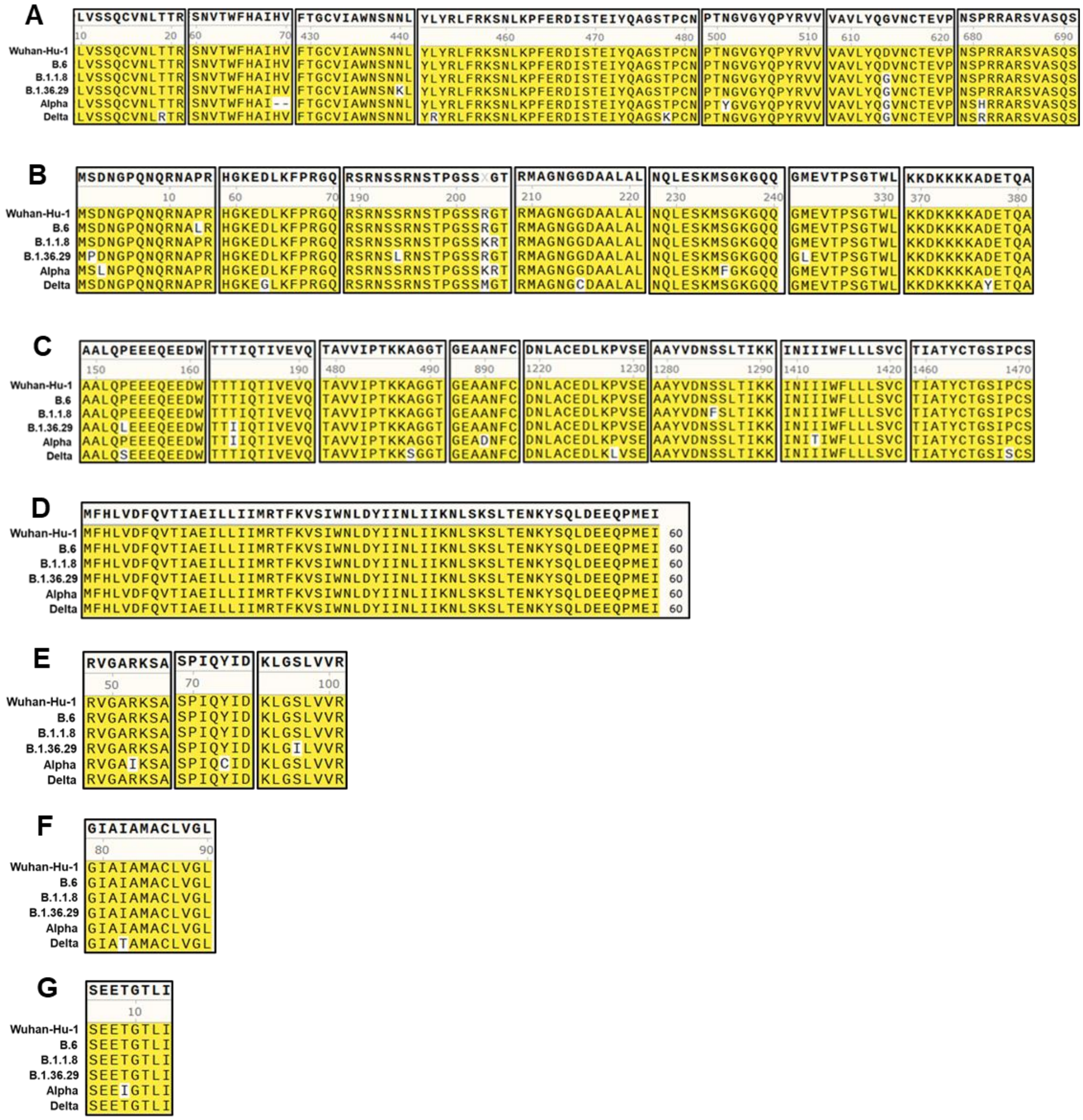

Supplementary Figure 10. Alignment of regions of various SARS-CoV-2 polypeptide sequences from the variants used in this study. (A: Spike; B: Nucleocapsid; C: Nsp3; D: ORF6; E: ORF8; F: Membrane; and G: Envelope)
